# Metropolitan Hospital Sunday Fund

**Published:** 1900-06-23

**Authors:** Alfred J. Newton

**Affiliations:** Lord Mayor. The Mansion House, London


### EDITOR'S LETTER-BOX.

[Our Correspondents are reminded that prolixity is a great bar to publication, and that brevity of style and conciseness of statement greatly facilitate early insertion.]

### METROPOLITAN HOSPITAL SUNDAY FUND.

Sir,—The many appeals which have recently emanated from the Mansion House in respect either of the war or the famine in India are being so liberally and bountifully met that I gravely hesitate to make yet another call upon the never-failing generosity of the inhabitants of London. I feel sure, however, that in reminding the public that next Sunday is "Hospital Sunday," when—for the twenty-eighth successive year—collections will be made in every place of worship for the hospitals and dispensaries of this vast metropolis, they will admit that even the exigencies of the war should not divert them from rendering that annual help which has become so all-important a part of the incomes of the medical charities. While the war has drawn from the hospitals of London the ready services of doctors and nurses who have rendered devoted and invaluable aid in dealing with the sick and wounded at the front, it has materially prejudiced the institutions financially through the urgent competition of the multitude of appeals associated with the campaign. But the progress of the war has effected no diminution of the numbers of sick poor for whom the hospitals of London labour unceasingly. Never was there a time in the history of the metropolis when there was greater need than now for the rendering of increased help towards the maintenance of these noble institutions, of whom some 200 owe their existence solely to the voluntary offerings of the benevolent public. When one reads that hundreds of beds are unused for want of funds, and that last year the expenditure on the hospitals exceeded the income by nearly a quarter of a million sterling, it seems time—even in a year of unparalleled stress and anxiety—to make a special effort in behalf of the medical charities of London. It is suggested that £100,000 might be raised this year for this object. Towards this Mr. George Herring has given £10,000 down and promised £10,000 more if £90,000 is raised. I trust the generous public, by their contributions on Hospital Sunday, will enable me to take advantage of this munificent offer. I would add that, as usual, donations from those unable to attend a place of worship on Sunday may be sent to me at the Mansion House for addition to the Fund.—I am, sir, your obedient servant,

Alfred J. Newton, Lord Mayor.

The Mansion House, London, June 19th, 1900.

